# The Texas Health College Evil Again

**Published:** 1897-03

**Authors:** 


### The Texas Health College Evil Again.

CANEY, TEXAS, Feb. 21, 1897.

*Editors Texas Medical Journal:*

We have here a man traveling from place to place with a show, examining and treating patients, and charging exorbitant prices for same, and upon examining the records I find he has a certificate from the Texas Health College.

Is he practicing lawfully? if not, what is my duty as a member of the examining board for this district?

Answer by mail and oblige, yours truly,

JAS. E. SIMONS, M. D.

---

OFFICE
TEXAS MEDICAL JOURNAL,
AUSTIN, TEXAS, Feb. 25, 1897.

*Dr. Jas. E. Simons, Member District Board Medical Examiners, Caney, Taxas:*

DEAR DOCTOR:—We are in receipt of your letter asking what is the duty of the examining board with reference to an itinerant, claiming the right to practice medicine (or, rather to hawk his nostrums on the street as an accompaniment to a "show"), by virtue of a so-called "diploma" issued by the alleged "Texas Health College." In reply we beg to say that it is a troublesome matter. We have had many similar letters to yours, and have consulted the attorney-general, who has promised that whenever we will bring to his knowledge a case where one of these diplomas is being used, hewill instruct the district attorney to present the party to the grand jury on a charge of fraud. He has written to several attorneys, at our instigation, but no one has been convicted, because, the attorney offers as an excuse that it will be necessary to prove that no such college exists or ever did exist, and that it takes money to get witnesses and affidavits, and that there is no money available for such purposes.

I talked yesterday with the member of the legislature from Coryell county (he is a lawyer), the county in which the so-called "Health College" is alleged to be situated, and asked what should be done to bring holders of these bogus diplomas to punishment? They have, technically, complied with our miserable excuse for a law; they have "registered a diploma from a chartered medical college;" still, it is a *fraud*, as no such college ever existed. The charter was granted, perhaps, in good faith, yet it is void, *per se*, because the purpose for which it was granted was never carried out. The legislature can do nothing, the members say, because, to revoke the charter, it is claimed, could not have a retro-active effect, nor invalidate or do away with existing "diplomas." The only way that the evil can be remedied, this gentleman and others tell us, is, to make a test case in one of the courts. The party if convicted will most likely, appeal, and thus get it before the highest court. The decision cannot be other than that the holder of the diploma is perpetrating fraud, and that decision would establish precedent.

In view of the inaction of the attorney-general, notwithstanding his promise to us to interest himself in the matter, and also interest and instruct local attorneys, the gentleman from Coryell county, and others, say that you should have the party arrested for practicing medicine in violation of the Penal Code. (You can refer to page 69 of the code in the office of any attorney.) The party will put in evidence his "diploma," and plead that he has complied with the law, having registered a "diploma," from a chartered medical college. Your lawyer should then enter a demurrer, and allege fraud, in that the charter was obtained on representations that were false, and its purposes never carried out, and is therefore, void. It will be necessary to obtain affidavits from citizens of Mound City that there is not, and never has been a medical college at that point, and this affidayit will not cost anything, as we believe that Dr. T. J. Smith, at Mound, or Dr. J. E. Webb, of Trenton, or Dr. J. S. Jones, of Gatesville, will cheerfully cooperate with you in this matter.

The Journal takes a deep interest in this matter and is anxious to see an example made of these fellows. It is so plainly a fraud that it would seem there ought not to be any difficulty in getting a conviction. Should you explain all the circumstances to the foreman of the grand jury, if he is a man of any intelligence, it seems to us the jury could devise some means of getting at the evil. We will gladly assist you in any way we can. Wishing you success, we are,

Very truly yours,

Daniel & Hudson.

